# Relapse Versus Reinfection of Recurrent Tuberculosis Patients in a National Tuberculosis Specialized Hospital in Beijing, China

**DOI:** 10.3389/fmicb.2018.01858

**Published:** 2018-08-14

**Authors:** Zhaojing Zong, Fengmin Huo, Jin Shi, Wei Jing, Yifeng Ma, Qian Liang, Guanglu Jiang, Guangming Dai, Hairong Huang, Yu Pang

**Affiliations:** ^1^National Clinical Laboratory on Tuberculosis, Beijing Key Laboratory of Drug Resistance Tuberculosis Research, Beijing Chest Hospital, Beijing Tuberculosis and Thoracic Tumor Research Institute, Capital Medical University, Beijing, China; ^2^Beijing Pediatric Institute, Beijing Children’s Hospital, Capital Medical University, Beijing, China

**Keywords:** tuberculosis, recurrence, relapse, reinfection, drug-resistant

## Abstract

Tuberculosis (TB) recurrence can result from either relapse of an original infection or exogenous reinfection with a new strain of *Mycobacterium tuberculosis* (MTB). The aim of this study was to assess the roles of relapse and reinfection among recurrent TB cases characterized by a high prevalence rate of drug-resistant TB within a hospital setting. After 58 paired recurrent TB cases were genotyped to distinguish relapse from reinfection, 37 (63.8%) were demonstrated to be relapse cases, while the remaining 21 were classified as reinfection cases. Statistical analysis revealed that male gender was a risk factor for TB reinfection, odds ratios and 95% confidence interval (OR [95% CI]: 4.188[1.012–17.392], *P* = 0.049). Of MTB isolates obtained from the 37 relapse cases, 11 exhibited conversion from susceptible to resistance to at least one antibiotic, with the most frequent emergence of drug resistance observed to be levofloxacin. For reinfection cases, reemergence of rifampicin-resistant isolates harboring double gene mutations, of codon 531 of *rpoB* and codon 306 of *embB*, were observed. In conclusion, our data demonstrate that relapse is a major mechanism leading to TB recurrence in Beijing Chest Hospital, a national hospital specialized in TB treatment. Moreover, male patients are at higher risk for reinfection. The extremely high rate of multidrug-resistant tuberculosis (MDR-TB) among reinfection cases reflects more successful transmission of MDR-TB strains versus non-resistant strains overall.

## Introduction

Tuberculosis (TB) is caused by bacteria of the *Mycobacterium tuberculosis* complex (MTBC) and is a major threat to public health worldwide (World Health Organization [WHO], 2016). Since the introduction of effective anti-TB drugs in the 1940s, most TB patients have been cured using combination drug treatment regimens. However, some patients still exhibit tuberculosis recurrence after treatment completion, a phenomenon which adds considerably to the burden of tuberculosis worldwide (Guerra-Assunção et al., 2015). Recurrence of TB can be due either to relapse of an original infection or to reinfection via exogenous infection by a new MTBC strain (Guerra-Assunção et al., 2015; Schiroli et al., 2015). In TB endemic areas, reinfection is the principle cause for observed recurrence rates (van Rie et al., 1999). In addition, a coexistent high incidence of human immunodeficiency virus has been demonstrated to exacerbate recurrence rates due to reinfection. Conversely, a high relapse rate may indicate unsuccessful treatment rather than recent TB transmission within the community (McIvor et al., 2017). Therefore, by differentiating between mechanisms underlying the two distinct types TB recurrence, relapse versus reinfection, ultimately more effective TB control strategies could be implemented to achieve better TB control (Guerra-Assunção et al., 2015).

The development of molecular genotyping methods for differentiating between MTB strains has made it possible to differentiate between the mechanisms giving rise to exogenous relapse and reinfection cases (Guerra-Assunção et al., 2015; Jagielski et al., 2016). However, the determination of reinfection rates may be influenced by different discriminatory powers of various genotyping methods (Jagielski et al., 2016). Ultimately, whole-genome sequencing would produce the greatest degree of discrimination of all methods, but would incur high costs (Bryant et al., 2013). As an alternative, most studies to date have employed IS6110-based restriction fragment-length polymorphism (RFLP) or mycobacterial interspersed repetitive unit-variable number of tandem repeat (MIRU-VNTR) analyses. Fortunately, these lower cost methods are powerful enough to effectively distinguish between organisms associated with reinfection and relapse episodes for a given patient (Shen et al., 2006, 2017).

After India and Indonesia, China has the third highest TB burden globally (World Health Organization [WHO], 2016), although during the past 20 years China has achieved great progress in halving its TB prevalence (Wang et al., 2014). Unfortunately, the epidemic of drug-resistant TB is threatening to destabilize TB control there (Zhao et al., 2012). Because TB relapse is usually associated with drug resistance (Cox et al., 2006), it is reasonable to predict that the emergence of drug-resistant TB will bias future recurrent TB cases away from reinfection rates toward greater relapse rates, as suggested by preliminary data from China regarding this issue (Shen et al., 2006, 2017; Yang et al., 2017). To provide additional evidence, the aim of this study was to assess the roles of relapse and reinfection among recurrent TB cases in a setting where approximate 35% of patients are infected with multidrug-resistant tuberculosis (MDR-TB). We also compared *in vitro* drug susceptibility test (DST) results between first and second TB episodes and investigated the influence of several risk factors on reinfection versus relapse recurrence rates.

## Materials and Methods

### Setting and Study Population

This retrospective cohort study utilized data within the TB patient database of the Beijing Chest Hospital, which is also a National Clinical Center on Tuberculosis in China. Approximate 10 thousands patients seek health care in this hospital each year, of whom 35% of patients are infected with MDR-TB. Eligible patients enrolled in this study met the following criteria: (1) completion of anti-TB treatment following the guidelines of National Tuberculosis Control Program; (2) exhibited two or more episodes of TB diagnosed in the period August 2011-July 2014 (within a minimum time interval of 12 months based on the date of the end of treatment of the first episode); (3) provided cultured MTB isolates from the first episode was used in this analysis. If the patients meeting the criteria had two or more recurrent episodes, the first was recruited in this analysis. To characterize patient MTB isolates, sputum specimens from TB patients were digested using the N-acetyl-L-cysteine-sodium hydroxide (NALC-NaOH) method. After 15 min of incubation, digested specimens were neutralized with sterile phosphate buffer (pH = 6.8) and centrifuged at 3000 *× g* for 15 min. Each pellet was resuspended in 2 mL of phosphate buffer and 0.5 mL of each suspension was inoculated onto Löwenstein-Jensen (L-J) medium, incubated at 37°C, then examined for mycobacterial growth twice weekly thereafter. Patient demographic characteristics and treatment histories were obtained by reviewing medical records. The study protocol was approved by the Ethics Committee of Beijing Chest Hospital, Capital Medical University and written informed consent was obtained from each participant prior to the study.

### Genotyping

Genomic DNA was extracted from fresh 4-week-old bacterial cultures grown on L-J medium as previous reported (Pang et al., 2012). Bacterial colonies were harvested from solid cultures and were transferred into microcentrifuge tubes containing 500 μL Tris-EDTA (TE) buffer. After inactivation in a 100°C water bath for 30 min, supernatants containing DNA templates were subjected to PCR amplification. The classical 24-locus MIRU-VNTR method was performed to genotype MTB isolates (Supply et al., 2006). To detect differences in repeat numbers, PCR products were examined by standard agarose gel electrophoresis (1.5% agarose), with visualization of DNA by fluorescence using GelRed dye^®^. The 100-bp DNA ladder (Transgene, Beijing, China) was loaded into wells of every fourth lane to serve as a size marker. Amplicon sizes were estimated using Quantity One software (Bio-Rad, Hercules, CA, United States). A reinfection case was defined by the occurrence of strains with different 24-locus MIRU-VNTR patterns at two or more loci between first and second TB episodes, as previously reported (Martín et al., 2011; Pang et al., 2015).

### Drug Susceptibility Testing

In order to investigate changes in drug susceptibility patterns between first and second TB episodes, we subcultured the isolates and used the alamarBlue assay to the minimal inhibitory concentrations (MICs) for MTB isolates from study patients (Zhang et al., 2014a). Briefly, bacterial colonies were scraped from the surface of L-J media. After vigorous vortexing for 1 min, each suspension was diluted to 1.0 McFarland turbidity value with 0.9% sodium chloride (normal saline). Each suspension was further diluted 1:20 in Middlebrook 7H9 broth supplemented with 10% OADC (oleic albumin dextrose catalase growth supplement). Next, 100 μL of this bacterial diluent was inoculated into each well of 96-well plates containing 100 μL of 7H9 broth containing serial dilutions of each antibiotic. After 7 days of incubation at 37°C, 70 μL of alamarBlue solution was added to each well for results interpretation. Color change was used to evaluate bacterial growth after incubation for an additional 24 h, followed by determination of MIC as defined as the lowest concentration of drug that inhibited color change from blue to pink. All experiments were performed in triplicate. MIC breakpoint concentrations were defined as 0.2 mg/L for isoniazid (INH), 1.0 mg/L for rifampicin (RIF), 5.0 mg/L for ethambutol (EMB), 1.0 mg/L for levofloxacin (LFX), 4.0 mg/L for amikacin (AMK), and 4.0 mg/L for capreomycin (CAP).

### PCR Amplification and DNA Sequencing

Genes known to confer drug resistance were studied here, including *rpoB* for rifampicin resistance (RIF), *katG* and *inhA* for INH resistance, *embB* for EMB resistance, *gyrA* and *gyrB* for fluoroquinolone (FQ) resistance, and *rrs* for resistance to second-line injectable drugs (**Supplementary Table S1**) (Chen et al., 2014). Gene fragments were amplified in a thermocycler as follows: 5 min at 94°C for initial denaturation; 35 cycles of 94°C for 1 min, 58°C for 1 min, and 72°C for 1 min; and 5 min at 72°C for final extension. PCR amplicons were purified using a PCR purification kit and sent to Rui Biotech Company (Beijing, China) for sequencing. DNA sequences were aligned to homologous sequences of the reference MTB H37Rv strain sequence found on the National Center for Biotechnology Information Server^1^.

### Statistical Analysis

Person chi-square test or Fisher’s exact test were used to compare proportions of patients in each subgroup or rates of drug resistance. Odds ratios (OR) and 95% confidence interval (95%CI) were calculated in the univariate analyses. The multivariate models were carried out by forward stepwise logistic regression procedures to identify factors that independently affected reinfection. In addition, changes in MIC values between first and second TB episodes were compared via *t*-test. The differences were declared as significant for *P-*value < 0.05. All statistical procedures were performed using SPSS version 17.0 software (SPSS Inc., Chicago, IL, United States).

## Results

### Samples Studied

**FIGURE 1:**
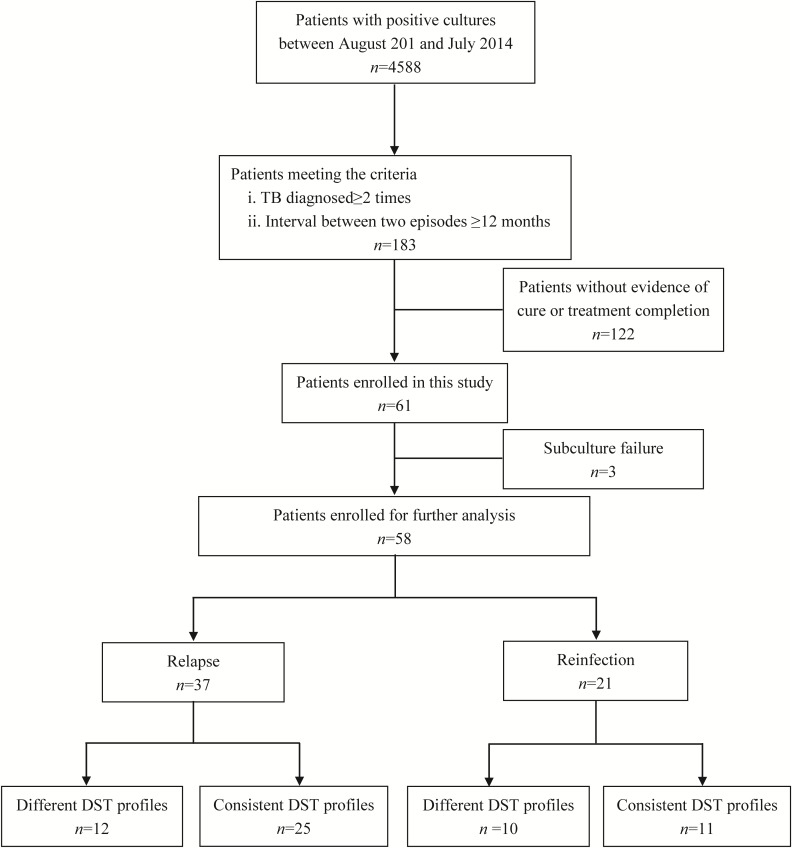
Patients Enrollment.

**FIGURE 2:**
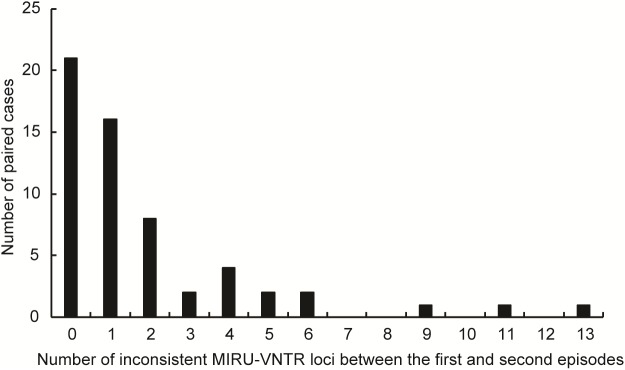
Distribution of recurrent cases with different MIRU-VNTR patterns. The abscissa represents the different number of copies of MIRU-VNTR loci in recurrence pairs. The ordinate represents the number of recurrence pairs with different number of copies of MIRU-VNTR loci.

Samples from total of 61 TB cases who were cured or had completed treatment at first visit in Beijing Chest Hospital produced positive cultures in post-treatment follow-up. Of these 61 recurrent cases, three were excluded due to subculture failure. The remaining 58 paired samples were further genotyped to distinguish between strains of TB versus reinfection strains (**Figure 1**). Comparisons of results of MIRU-VNTR patterns for paired samples collected from first and second TB episodes are shown in **Figure 2** and **Supplementary Figure S1**. The results showed that 21 (36.2%, *n* = 21/58) pairs of isolates exhibited consistent MIRU-VNTR patterns, while 16 pairs (27.6%, *n* = 16/58) exhibited different patterns between episodes after testing of only one locus. Therefore, 37 (63.8%) of 58 total recurrent TB cases were classified as relapse cases. In contrast, the other 21 pairs of isolates exhibited different 24-locus MIRU-VNTR patterns for two or more loci between the first and second episode and were therefore classified as reinfection cases (Velayutham et al., 2018).

### Differences in Characteristics Between Relapse and Reinfection Cases

We further compared characteristics between relapse cases and reinfection cases. Overall, there were significantly more reinfection cases among male versus female patients, indicating that male gender is a risk factor for TB reinfection (OR [95% CI]: 4.188[1.012–17.392], *P* = 0.049). In contrast, ages, drug resistant profiles, the presence of comorbidities and the timing of a recurrent episode had no influence on percentage of relapse cases (**Table 1**).

**Table 1:** Demographic and clinical characteristics of tuberculosis patients enrolled in this study.

Characteristics	Reinfection	Relapse	Total	Odds ratios	Adjusted odds	*P*-value
	*n* = 21 (%)	*n* = 37 (%)	*n* = 58 (%)	(95% CI)^b^	ratios (95% CI)	
*Gender*						
Male	18(85.7)	22(59.5)	40(69.0)	4.091(1.022–16.380)	4.188(1.012–17.392)	0.049
Female	3(14.3)	15(40.5)	18(31.0)	Ref.	Ref.	
						
*Age group (years)*						
<25	1(4.8)	2(5.4)	3(5.2)	1.400(0.103–19.012)		
25–44	5(23.8)	14(37.8)	19(32.8)	Ref.		
> 44	15(71.4)	21(56.8)	36(62.1)	2.000(0.592–6.756)		
						
Cavity						
Yes	9(42.9)	18(48.6)	27(46.6)	0.791(0.269–0.430)		
No	12(57.1)	19(51.4)	31(53.4)	Ref.		
						
*Comorbidity*						
Diabetes	1(4.8)	10(27.0)	11(19.0)	0.117(0.013–1.015)	0.131(0.015–1.149)	0.067
Liver disease	2(9.572)	4(10.8)	6(10.3)	0.588(0.965–3.623)		
Others	1(4.8)	3(8.1)	4(6.9)	0.392(0.037–4.132)		
No	17(81.0)	20(54.1)	37(63.8)	Ref.	Ref.	
						
*Drug resistant profiles*						
MDR^a^	13(61.9)	25(67.6)	38(65.5)	0.639(0.211–1.930)		
Non-MDR	8(38.1)	12(32.4)	20(34.5)	Ref.		
						
*Time of recurrent episode*						
≤2 years	9(42.9)	19(51.4)	28(48.3)	0.710(0.241–0.242)		
>2 years	12(57.1)	18(48.6)	30(51.7)	Ref.		

*^a^MDR, multidrug-resistant.*

*^b^CI, confidence interval.*

### Differences in Drug Susceptibility Profiles Between First and Second TB Episode

**FIGURE 3:**
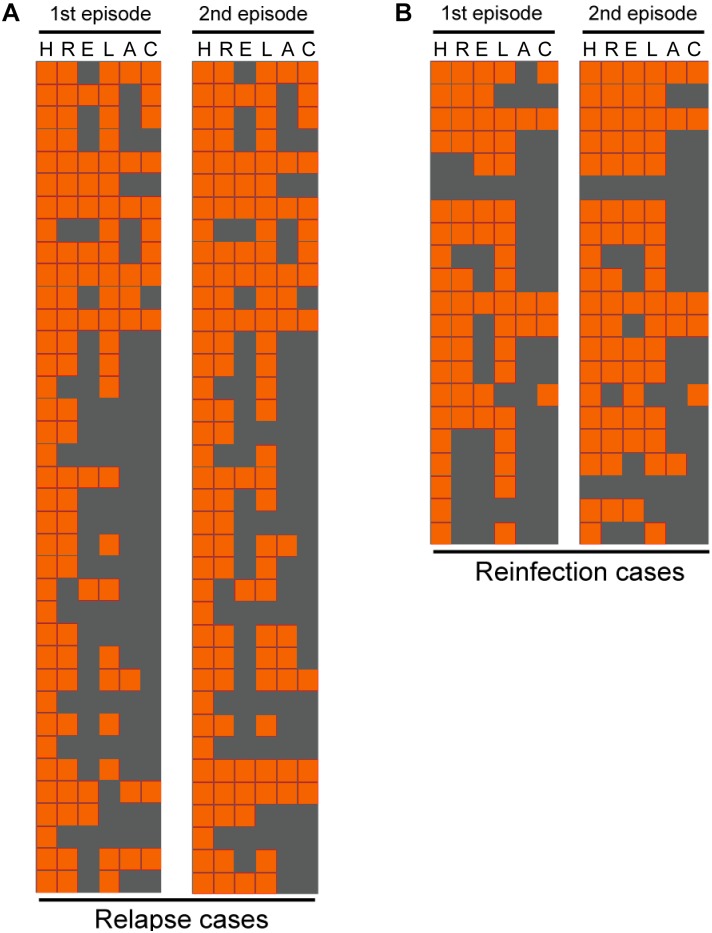
Comparison of drug susceptibility profiles between the first and second episode. Each line represents the drug susceptibility results from two episodes of each patient. The results are shown in an orange-gray color scale, where orange represents resistant to the corresponding drug and gray represents susceptible to the corresponding drug. I, isoniazid; R, rifampicin; E, ethambutol; L, levofloxacin; A, amikacin; C, capreomycin.

Comparison of drug susceptibility profiles of recurrent cases between the first and second episode were summarized in **Figure 3**. As shown in **Figure 1**, of MTB strains from 37 relapse cases, 12 (32.4%, *n* = 12/37) exhibited different DST profiles between the first and second TB episode. Of these 12 paired samples, 11 exhibited changes from sensitivity to resistance toward at least one antibiotic agent, while one previously AMK-resistant strain was susceptible to AMK during the second episode. The most frequent observation, emergence of drug resistance to LFX, was observed for 6 of 11 relapse strains that had been sensitive to LFX in the first episode (54.5%, *n* = 6/11). This result suggests that LFX resistance was acquired by the original strain, with similar results observed for resistance to AMK (36.4%, *n* = 4/11), CAP (18.2%, *n* = 2/11), and EMB (18.2%, *n* = 2/11) shown in **Table 2**. Meanwhile, analysis of drug-susceptibility results of isolates from reinfection patients revealed that 8 second-episode isolates (38.1%, *n* = 8/21) gained resistance to at least one drug as compared with corresponding first-episode isolates.

**Table 2:** Differences in drug susceptibility profiles between the first and second episode.

Drug	Relapse *n* = 37(%)			Reinfection *n* = 21(%)		
	S^a^ →R^b^	R→S	No change	S→R	R→S	No change
INH	0(0)	0(0)	37(100)	1(4.8)	1(4.8)	19(90.4)
RIF	0(0)	0(0)	37(100)	4(19.0)	1(4.8)	16(76.2)
EMB	2(5.4)	0(0)	35(94.6)	4(19.0)	0(0)	17(81.0)
LFX	6(16.2)	0(0)	31(83.8)	1(4.8)	1(4.8)	19(90.4)
AMK	4(10.8)	1(2.7)	32(86.5)	2(9.6)	0(0)	19(90.4)
CAP	2(5.4)	1(2.7)	34(91.9)	0(0)	0(0)	21(100)
Total	11(29.7)	1(2.7)	25(67.6)	8(38.1)	2(9.5)	11(52.4)

*^a^S, sensitive.*

*^b^R, resistant.*

We further analyzed genetic mutations in order to rationalize these observed changes in drug resistance profiles of paired isolates, including four isolates for INH, 10 for RIF, 12 for EMB, 16 for LFX and 14 for second-line injectable drugs. All DNA fragments amplified from isolates were analyzed by DNA sequencing and the results are summarized in **Table 3**. For relapse cases, changes in LFX susceptibility were mainly due to a mutation in codon 94 of *gyrA* (50%, *n* = 3/6) that resulted in amino acid substitution from Asp to Gly. Meanwhile, mutants with an Ala1401Gly mutation in the *rrs* gene partially accounted for the appearance of AMK (40%, *n* = 2/5) and CAP (66.7%, *n* = 2/3) resistance. For reinfection cases, reemergence of RIF-susceptible isolates followed again by emergence of RIF-resistant (RR) isolates was explained by mutations in codon 531 of the *rpoB* gene. Interestingly, all reemerged RIF-resistant isolates also had the Met306Val mutations in embB gene.

**Table 3:** Genetic mutations identified MTB isolates with different drug susceptibility profiles between the first and second episodes.

Drug	Gene	Relapse		Reinfection	
		S→R	R→S	S→R	R→S
INH	*InhA*	–	–	WT→C -15 T(1)	No change (1)
	*KatG*	–	–	WT→AGC315ACC (1)	AGC315ACC→WT (1)
RIF	*RpoB*	–	–	WT→TCG531TTG (3)	WT→TCG531TTG (1)
				WT→CAC526TAC (1)	
EMB	*embB*	WT→ATG306GTG (2)	–	WT→ATG306GTG (4)	–
LFX	*GyrA*	WT→GAC94GGC (3)	–	WT→GAC94GCC (1)	No change (1)
		GAC89AAC→GAC94GGC (1)			
		No change (2)			
	*GyrB*	WT→GCG543GTG (1)	–	No change (1)	No change (1)
		No change (5)			
AMK	*Rrs*	WT→A 1401 G (2)	No change (1)	WT→ A1401G (1)	–
		WT→A 1368 G (1)		No change (1)	
		No change (1)			
CAP	*Rrs*	WT→A 1401 G (2)	No change (1)	–	–

## Discussion

The use of a standard genotyping method has provided new insights into the role of relapse and reinfection in recurrent TB cases (Jagielski et al., 2016). In this study, 63.8% (*n* = 37/58) of recurrent TB cases resulted from reactivation of MTB infection (relapse) from a previous TB episode. This is in contrast to findings from regions of high TB burden, where reinfection is the major mechanism leading to TB recurrence (Lambert et al., 2003). Meanwhile, because there is strong evidence that drug resistant bacteria, especially MDR-TB, contribute to high relapse rates after successful treatment (Cox et al., 2006), inadequate treatment regimens for MDR-TB patients may largely explain high relapse rates. For the treatment of MDR-TB, second-line injectable drugs and FQs constitute the cornerstone of most therapeutic regimens (Di Perri and Bonora, 2004). However, these antimicrobial agents are bactericidal only against rapidly replicating mycobacteria, with little or no activity against dormant bacilli. Subsequently, the survival of dormant MDR-TB has no doubt increased the risk of TB relapse in this population during follow-up.

Notably, we found that male patients were at higher risk for reinfection as compared to female patients. On one hand, this observation may result from more frequent social communication between men that leads to an increased risk of TB overexposure, especially for patients living in settings with high TB prevalence. On the other hand, TB detection is two times higher in men than women in most countries, reflecting the fact that men are more prone to TB infection (vom Steeg and Klein, 2016). Notably, recent animal models suggest an increased risk for disease progression in males (Dibbern et al., 2017). Therefore, male-female differences in combating TB may play an important role in the high occurrence rate of reinfection in male patients.

Another interesting finding of this study was that emergence of LFX resistance was the most frequently observed change in drug resistance between episodes, with 54.5% (*n* = 6/11) of relapse cases observed to convert to LFX-resistance by the second episode. Although the exact reasons remain unknown, there are several potential explanations for this high LFX resistance rate. First, due to its potent activity against MTB for relatively low cost, LFX is widely used to treat patients infected with drug-resistant TB (Zhang et al., 2014a). In line with previous observations, almost 27.6% (*n* = 16/58) of patients in this study had received regimens containing LFX, whereas another later-generation FQ, moxifloxacin (MFX), had been used as an alternative treatment for the other 53.4% (*n* = 37/58) of MTB patients. Second, FQs are often used for treating diverse types of infections and a number of recent studies have demonstrated that abuse of FQs may correlate with the high FQ-resistance rate of MTB isolates in China (Pang et al., 2017). Consequently, high exposure to FQs may be associated with accumulation of adaptive mutations conferring FQ resistance, thus leading to the emergence of resistant bacteria as a predominant population. Third, as an important action of FQs on bacteria, FQs are effective inducers of the SOS DNA repair system, which is associated with an elevated mutation rate (Ysern et al., 1990). Although experimental evidence is lacking, we therefore hypothesize that the elevated mutation rate might be another important factor responsible for observed increases in FQ resistance.

In addition to relapse cases, our analysis of reinfection cases allowed us to identify changes in drug susceptibility results between paired first and second TB episode isolates. Remarkably, reinfection episodes were more likely to exhibit resistance to both RIF and EMB, with one-fifth of MTB isolates from reinfection cases exhibiting this resistance profile. Notably, all isolates exhibiting variations of RIF susceptibility were MDR-TB and harbored genetic mutations in codon 306 of *embB*. This result echoes previous findings that nucleotide acid substitution within codon 306 of *embB* is a potential molecular marker for predicting MDR phenotype (Zhang et al., 2014b; Cuevas-Córdoba et al., 2015). More importantly, 61.9% (*n* = 13/21) of reinfection cases were MDR cases, significantly higher than the average level of MDR resistance (8.3%) in China (Zhao et al., 2012). Although only a relatively small number of strains were assayed here, increases in multidrug resistance would be further evidence that recent successful transmission of MDR-TB strains is driving the MDR tuberculosis epidemic in Shanghai, China (Yang et al., 2015). In fact, this situation may even be more serious in rural areas of China, where TB patients do not have access to drug susceptibility testing due to poor laboratory resources. In addition, the extremely high rate of MDR-TB among reinfection cases indicates that the MDR-TB burden in China may be an underestimate, as all patients enrolled in the previous national surveillance study came from the Center for Disease Control (CDC) sector rather than the TB hospital sector, for which MDR-TB prevalence of the latter (35%) is significantly higher than that of the former (8.3%) (Zhao et al., 2012). Therefore, surveillance focused on prevalence of drug-resistant TB within the TB hospital sector is urgently needed.

We must acknowledge several obvious limitations of this study. First, the 24-locus MIRU-VNTR typing method was used to distinguish between relapse and exogenous reinfection cases in spite of its relatively poor discriminatory power as compared with whole-genome sequencing. The results may therefore overestimate the prevalence of relapse versus reinfection in this study. Second, despite the enrollment of patients who all met the criteria throughout the study period, the small number of patients and unbalanced sample size between groups may limit the overall significance of our study conclusion. Third, the number of recurrent TB cases detected here was relatively low due to negative culture results and loss of recurrent patients due to migration to other health sectors. Thus, it is difficult to estimate the true recurrence rate within a hospital and this shortcoming may bias the conclusions. Finally, although several investigations have demonstrated that the TB recurrence is associated with poor clinical outcomes, the final outcomes of recurrent cases in our study were not determined due to the long follow-up period necessary to collect this data.

## Conclusion

Our data demonstrate that relapse is a major mechanism leading to TB recurrence in patients under the care of a national tuberculosis specialized hospital. Moreover, male patients have a higher risk for reinfection than do female patients. Meanwhile, emergence of LFX resistance in the second TB episode was most frequently observed during relapse, while reinfection episodes more likely exhibited emergence of RIF and EMB resistance. These results collectively illustrate that greater understanding of the differences between relapse and reinfection types of recurrent TB can provide important information for the design of more effective downstream TB control interventions. Such interventions are urgently needed to counter extremely high rates of MDR-TB among reinfection cases that reflects highly successful MDR-TB transmission, underscoring the immediate need for better TB control strategies in China.

## Author Contributions

ZZ, FH, JS, YP, and HH designed the study. ZZ, FH, JS, WJ, YM, and QL performed the experiments. ZZ, FH, JS, GJ, and GD interpreted the data. ZZ, FH, JS, YP, and HH wrote the manuscript. All authors approved the final version of the manuscript.

## Conflict of Interest Statement

The authors declare that the research was conducted in the absence of any commercial or financial relationships that could be construed as a potential conflict of interest.

## Footnotes

1 www.ncbi.nlm.nih.gov
