# Supplementary material for: Relapse Versus Reinfection of Recurrent Tuberculosis Patients in a National Tuberculosis Specialized Hospital in Beijing, China
Source: Front Microbiol. 2018 Aug 14;9:1858. doi: 10.3389/fmicb.2018.01858 (PMC6102324; doi:10.3389/fmicb.2018.01858)
Supplement: Supplementary file 1 [file Table_1.DOCX]

**Supplemental Table 1 Primers used in this study for amplification and sequencing**

| **Primer name** | **Sense** | **Sequence (5′ to 3′)** | **Fragment size (bp)** | **Length∗ (bp)** |
| --- | --- | --- | --- | --- |
| *inh*A | F | TGCCCAGAAAGGGATCCGTCATG | 23 | 455 |
|  | R | ATGAGGAATGCGTCCGCGGA | 20 |  |
| *kat*G | F | AACGACGTCGAAACAGCGGC | 20 | 455 |
|  | R | GCGAACTCGTCGGCCAATTC | 20 |  |
| *rpo*B | F | CGACCACTTCGGCAACCG | 18 | 351 |
|  | R | TCGATCGGGCACATCCGG | 18 |  |
| *emb*B | F | CTGACCGACGCCGTGGTGATAT | 22 | 490 |
|  | R | TGAATGCGGCGGTAACGACG | 20 |  |
| *gyr*A | F | TCGACTATGCGATGAGCGTG | 20 | 415 |
|  | R | GGTAGCACCGTCGGCTCTTG | 20 |  |
| *gyrB* | F | CCGCTGTGATCTCGGTGAAG | 20 | 775 |
|  | R | AGACCCTTGTACCGCTGAATG | 21 |  |
| *rrs* | F | GTCAACTCGGAGGAAGGTGG | 19 | 516 |
|  | R | GTCCGAGTGTTGCCTCAGG | 20 |  |
