# Supplementary figures and images for: Relapse Versus Reinfection of Recurrent Tuberculosis Patients in a National Tuberculosis Specialized Hospital in Beijing, China
Source: Front Microbiol. 2018 Aug 14;9:1858. doi: 10.3389/fmicb.2018.01858 (PMC6102324; doi:10.3389/fmicb.2018.01858)

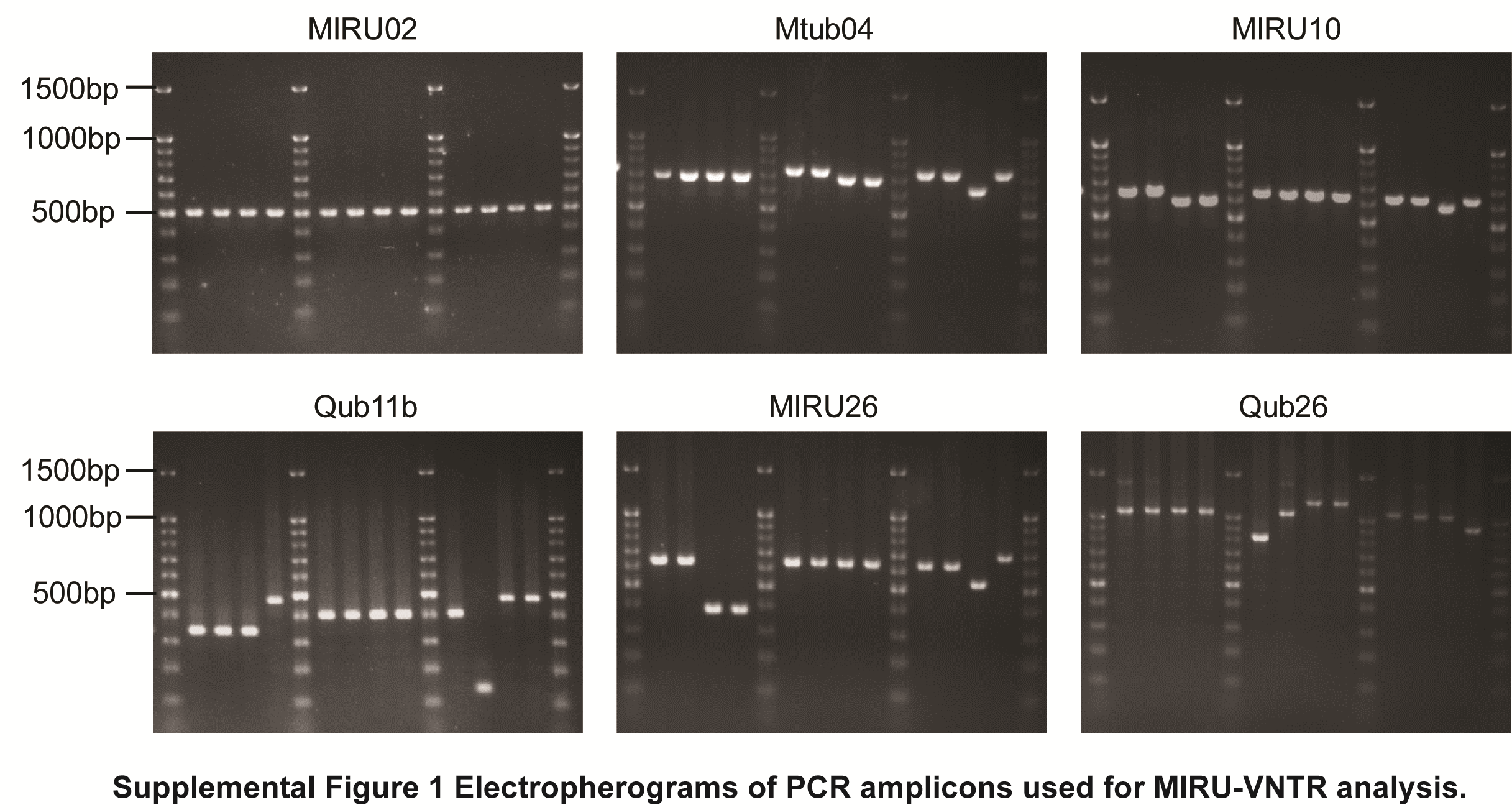

Supplement: Supplementary file 2 [file Image_1.TIF]
